# Behavioral Characterization of Mouse Models of Neuroferritinopathy

**DOI:** 10.1371/journal.pone.0118990

**Published:** 2015-02-17

**Authors:** Sara Capoccia, Federica Maccarinelli, Barbara Buffoli, Luigi F. Rodella, Ottavio Cremona, Paolo Arosio, Francesca Cirulli

**Affiliations:** 1 Department of Cell Biology, Section of Behavioral Neuroscience, Istituto Superiore Di Sanità, Rome, Italy; 2 Department of Molecular and Translational Medicine, Section of Biotechnologies, University of Brescia, Brescia, Italy; 3 Department of Clinical and Experimental Sciences, Section of Anatomy and Physiopathology, University of Brescia, Brescia, Italy; 4 Università Vita-Salute San Raffaele & Centro di Imaging Sperimentale, Istituto Scientifico San Raffaele, Milano, Italy; The Pennsylvania State University Hershey Medical Center, UNITED STATES

## Abstract

Ferritin is the main intracellular protein of iron storage with a central role in the regulation of iron metabolism and detoxification. Nucleotide insertions in the last exon of the ferritin light chain cause a neurodegenerative disease known as Neuroferritinopathy, characterized by iron deposition in the brain, particularly in the cerebellum, basal ganglia and motor cortex. The disease progresses relentlessly, leading to dystonia, chorea, motor disability and neuropsychiatry features. The characterization of a good animal model is required to compare and contrast specific features with the human disease, in order to gain new insights on the consequences of chronic iron overload on brain function and behavior. To this aim we studied an animal model expressing the pathogenic human FTL mutant 498InsTC under the phosphoglycerate kinase (PGK) promoter. Transgenic (Tg) mice showed strong accumulation of the mutated protein in the brain, which increased with age, and this was accompanied by brain accumulation of ferritin/iron bodies, the main pathologic hallmark of human neuroferritinopathy. Tg-mice were tested throughout development and aging at 2-, 8- and 18-months for motor coordination and balance (Beam Walking and Footprint tests). The Tg-mice showed a significant decrease in motor coordination at 8 and 18 months of age, with a shorter latency to fall and abnormal gait. Furthermore, one group of aged naïve subjects was challenged with two herbicides (Paraquat and Maneb) known to cause oxidative damage. The treatment led to a paradoxical increase in behavioral activation in the transgenic mice, suggestive of altered functioning of the dopaminergic system. Overall, data indicate that mice carrying the pathogenic FTL498InsTC mutation show motor deficits with a developmental profile suggestive of a progressive pathology, as in the human disease. These mice could be a powerful tool to study the neurodegenerative mechanisms leading to the disease and help developing specific therapeutic targets.

## Introduction

Iron is essential for cell viability, including electron transport in the respiratory chain, catabolism of neurotransmitters and neuronal development and myelination in the central nervous system [1–4]. Brain iron levels are not detectable at birth and start accumulating later during development. The causes of iron accumulation during aging are still unclear but could be related to dysfunction of blood brain barrier [5, 6] and to apoptosis and cellular damage [7]. Iron homeostasis must be finely regulated because iron is essential but also potentially toxic. In fact when in excess, iron can catalyze the formation of highly reactive free radicals via Fenton−like reactions. High iron deposition occurs in most neurodegenerative disorders including Alzheimer’s and Parkinson’s diseases, amyothropic lateral sclerosis, prion disease and a pool of genetic disorders collectively identified as Neurodegeneration with Brain Iron Accumulation (NBIA) [8, 9]. In these pathologies, local alterations of iron levels and/or of proteins involved in iron metabolism (particularly ferritins) have been reported, but it is unclear whether this is a causative factor or whether it represents a consequence of the degenerative processes.

Ferritins are ubiquitous iron storage molecules that play a central role in the regulation of iron metabolism and detoxification. Cytosolic ferritins are 24-mer heteropolymers composed of tissue-specific proportions of H- and L-chains, while mitochondrial ferritins are homopolymers [10]. The ferritins bind and incorporate iron in their large cavity by complex reactions that involve Fe(II) oxidation catalyzed by the ferroxidase center in the H subunit, followed by iron hydrolysis and mineralization facilitated by acidic residues of L-chains [11]. In this manner ferritins control Fe(II) availability and reduce radical oxygen species (ROS) production. DNA variations in the H−ferritin gene are very rare and its deletion in knockout mice is lethal at the embryonic stage [12]. In contrast, DNA variations in L-ferritin gene (FTL) are more common, and nucleotide insertions that modify the C-terminal region cause movement disorders named neuroferritinopathies, that are inherited with dominant transmission. Neuroferritinopathy was discovered in 2001 in a large pedigree in England, and now there are more than seventy cases found in Europe, America and Asia. It is a late-onset movement disorder characterized by neurodegeneration and abnormal brain iron accumulation (NBIA).

Nine pathogenic mutations of the FTL gene have been reported so far. One is a missense mutation in the third exon, whose pathogenicity is questioned [13]. All the other ones are one- or multiple-nucleotide insertions in the fourth exon that determine a frameshift and alterations of the C-terminus region involved in the four-fold symmetry channel. The largest study investigated subjects with the original 460InsA mutation, the clinical phenotypes was characterized in 40 English patients [14, 15]. The 498InsTC genotype was reported in 7 patients with symptoms similar to those of the 460InsA type [16, 17]. The genotype 458dupA was described in 4 French patients [18]. The mutation 469–484dup16nt was found in a single subject in Japan [19] and in one in Italy [20]. The 442dup4bp genotype was described in 7 Japanese patients [21], and the 442InsC found in two cases [22]. The last two mutations are a duplication of a thymine in position 468 described in a single France patient and the mutation 468_483dup16bp found in a Japanese family [23, 24]. The genotypes showed a complete penetration, with heterogeneity of clinical manifestations that were difficult to attribute to the different genotypes [25]. The clinical onset of neuroferritinopathy is around 40 years. The majority of patients presented focal onset chorea or focal dystonia and reported a family history of movement disorders [14]. Serum ferritin level is generally low.

Magnetic resonance imaging (MRI) studies show cerebral and cerebellar atrophy and abnormal signals in the basal ganglia [26, 27]. The disease progresses relentlessly and becomes generalized in 5–10 years, leading to aphonia, dysphagia and motor disability, with cognitive dysfunction in a late feature [9, 14]. In these patients, both neurons and glial cells show accumulation of intranuclear and intracytoplasmic inclusion bodies containing wild type and mutated ferritin light chain, ferritin heavy chain and iron. These aggregates are more abundant in the caudate nucleus, putamen and globus pallidus, but also present throughout the gray and white matter of the brain. Signs of neurodegeneration − including axonal swelling, cysts formation and increased immunoreactivity for neurofilaments, ubiquitin and tau protein − are most evident in the areas where inclusion bodies are formed [17, 22, 27]. Basal ganglia are involved in motor control and are particularly rich in iron in adult age [28]. Excessive cellular iron, associated with increased oxidative stress and the generation of reactive oxygen species (ROS), has damaging effects on many cellular processes [10, 29, 30] and can interfere with both the direct and the indirect pathway of movement contributing to the pathophysiological alterations in neurodegenerative disease, such as Parkinson and Neuroferritinopathy [31]. In particular, striatal-thalamo-cortical dysfunction induced by nigral dopaminergic degeneration has been demonstrated to be responsible for the occurrence of rigidity and bradykinesia [32, 33], while cerebellar-thalamic-cortical dysfunction may be involved in the occurrence of tremor in Parkinson’s disease [33, 34].

The effect of the pathogenic mutations is to disrupt important amino acid interactions for the correct folding around the hydrophobic channel whose integrity is necessary for protein functionality [35]. The biological effects of the mutations have been studied in cellular models, showing that they alter iron homeostasis with an increase of free iron and ferritins accompanied by higher sensitivity to hydrogen peroxide and oxidative damage [36]. This is consistent with a dominant negative effect of the mutations on ferritin functionality [35]. Animal models would be very important to characterize the disorder. A transgenic mouse model expressing a human cDNA carrying the FTL498InsTC mutation driven by the mouse prion protein (MoPrP) Xho promoter [37], was generated and characterized by Vidal and colleagues [38]. These mice showed alteration in the intracellular localization of ferritin already at the earliest ages examined (4 months), followed by an age-dependent progressive intracellular accumulation [38]. These authors described a progressive neurological phenotype with a significant decrease in motor performance, and a shorter lifespan [39]. We studied a new transgenic mouse model, similar to that described by Vidal, expressing the same pathogenic mutant of human ferritin-L chain but under the phosphoglycerate kinase (PGK) promoter, that has recently been characterized [40]. As it is similar to the one described by Vidal [38] it was named Tg.

## Materials and Methods

### Animals

A transgenic FVB-mouse model expressing the pathogenic human FTL mutant 498InsTC under the PGK promoter has been generated by pronuclear microinjection [40]. The FVB/N strain is recommended for transgenic-mice generation [41], but it shows a poor performance in several behavioral tests [42]. For **this** reason, the Tg mice were backcrossed for 7 generations in the C57BL/6J background.

Mice were group-housed in the same room provided by air conditioning (temperature 21 ± 1°C, relative humidity 60 ± 10%), in transparent Plexiglas cages (29cm × 12cm × 14cm), under a reversed 12/12 hr light/dark cycle (lights off 08:00–20:00). Pellet food (standard diet Altromin-R, Rieper, Italy) and tap water were continuously available. Animal handling and experimental procedures were performed in accordance with the EC guidelines (EC Council Directive 86/609 1987) and with the Italian legislation on animal experimentation (Decreto L.vo 116/92), and the project was approved Italian Ministry of Health.

## Experimental Procedures

### Quantitative qRT-PCR

Mice **(3 male and 3 female 12-month-old Tg-mice)** were anesthetized with Avertine (23 μl/gr mouse; Sigma) and perfused with a physiological saline solution containing 2% heparin; brain samples were then removed, dissected and immediately frozen. RNA was recovered from the different areas of the mouse brain with TRI Reagent (Sigma Aldrich) according to the manufacturer’s instructions. Reverse transcription was performed using 2μg RNA, oligo-dT, and Improm Reverse Transcriptase (Promega) in 20μl. Samples of 2μl were used for quantitative reverse-transcription polymerase chain reaction (qRT-PCR) assay, using iTaq Universal SYBR Green (BioRad) according to the manufacturer’s instructions. Extracted levels were quantified with qRT-PCR in a relationship to Hprt1 mRNA. Primers used to amplify humFTL were 5’-CCTAGATGAGGAAGTGAAGCTT-3’ and 5’- AGAGATACTCGCCCAGCCC-3’, and for HPRT1 were 5’CTGGTTAAGCAGTACAGCCCCAA-3’ and 5’-CAGGAGGTCCTTTTCACCAGC-3’.

### Histochemistry and immunohistochemistry

Analyses were performed at different ages: 6, 12 and 18 months-old (groups of at least 3 Tg and 3 WT-mice per age). Mice were anesthetized with Avertine (23 μl/gr mouse; Sigma) and perfused transcardially with paraformaldehyde 4% (Biooptica, Milan, Italy); the organs were removed and stored at -80°C (for histochemical analysis) or embedded in paraffin (for immunohistochemical analysis).

For histochemistry, brains were cut serially in 40 μm-thick sections using a cryostat. To show the presence of iron deposits, sections were stained with Prussian blue followed by 3, 3’-diaminobenzidine (DAB) for enhanced Perl’s reaction and counterstained with nuclear fast red 0.01% (Merck). Quantitative analysis of iron deposits (brown deposits) was performed in randomly selected cortical areas. Digitally fixed images (standardized arbitrary area) for each section (5 sections/animal) was evaluated using an optical microscope (Olympus BX50, Olympus, Hamburg, Germany) at a final magnification of 20X. The percentage of brown staining/field was calculated using a digital color video camera equipped with an image analysis program (Image Pro-Plus, Milan, Italy). The fields were randomly selected by two investigators unaware of the animal group assignment. Statistical analysis was performed using Anova One-Way Test, corrected by Bonferroni with P<0.05 considered as significant.

For immunohistochemistry, brains were cut serially in 7 μm-thick sections using a microtome. Human FTL expression was detected using a mouse monoclonal antibody named LF03 (1:100 for 2 hr at 21°C), previously described [43]. The reaction product was visualized using hydrogen peroxide and DAB (5mg/10ml PBS) as chromogen. Negative control for immunohistochemical reaction was performed by omitting the primary antibody. Quantitative analysis of LF03 immunopositivity was performed evaluating the percentage of brown staining in standardized area using an optical microscope (Olympus, Germany) equipped with an image analyzer (Image Pro-Plus, Milan, Italy).

### Iron-chelator treatment

To reduce the iron load, groups of 3 mice, 12-month-old, were treated for 3 weeks with the oral iron-chelator Deferiprone. It was dissolved in sweetened drinking water to 1mg/ml, and we controlled that water consumption, with and without the chelator, was the same, about 7ml per day.

### Neurotoxicant drugs treatment

The FVB/N strain is recommended for transgenic generation (Taketo, Schroeder et al. 1991), but it shows a poor performance in several behavioral tests (Mineur and Crusio 2002). Therefore we thought to use these aged mice to verify differences in response to environmental neurotoxicant exposures, which are hypothesized to increase the risk of developing neurodegenerative disorders (McCormack, Thiruchelvam et al. 2002; Thiruchelvam, McCormack et al. 2003). To test this hypothesis, we administered the herbicide Paraquat (PQ) and the fungicide Maneb (MB) to 21-month-old WT and Tg-mice. The treatment with PQ+MB have been previously shown to cause damage of the nigrostriatal system and to lead to neuronal death (McCormack, Thiruchelvam et al. 2002; Murray-Kolb, Welch et al. 2003; Thiruchelvam, McCormack et al. 2003). Transgenic-mice (n = 5) and wild-type (n = 5) littermates were given a single IP injection of the herbicide Paraquat (10mg/Kg dissolved in saline) and one of the fungicide Maneb (30mg/Kg dissolved in vegetable oil). The mice were tested in the Open Field and Beam Walking tests 24 h after the treatments.

### Behavioral Tests

Transgenic C57-mice and their wild-type littermates were studied at different ages with different behavioral tests. Mice were divided in three groups of different age: a) young mice, 2-month-old (n = 7 male and 7 female WT; n = 8 male and 8 female Tg); b) adult mice, 8-month-old (n = 18 male and 12 female WT; n = 15 male and 13 female Tg); c) aged mice, 18-month-old (n = 6 male and 5 female WT; n = 5 male and 5 female Tg). Motor coordination and balance were assessed using Beam Walking and Footprint test. Each behavioral test was video recorded and scored with specific software (Observer, Noldus).

### Open Field Test

Spontaneous locomotor activity was measured using a standard Open Field test [44]. Mice were individually placed in the center of a cubic arena (freshly cleaned open field box 44cm × 44cm × 44cm) made of grey Plexiglas and allowed to freely explore for 20 min with a novel object in the center of the arena during the last 5 minutes (a 50 ml glass beaker); The open-field box was ideally divided into 25 squares. When data were analyzed, each session was subdivided in 4 time blocks (tb) and the latency, frequency and duration of locomotion (the number of lines crossed or squared entered with four paws), exploratory activity (sniffing and rearing), risk assessment (stretched-attend posture—SAP), self-directed behaviors (self-grooming) and object exploration (sniffing and touching) were **scored [44–46]**. Behaviors were defined as follows:

- crossing: crossing the square limits with all paws;
- sniffing: self-explanatory;
- rearing: standing on the hind paws;
- stretched-attend posture: exploratory posture in which the body is stretched forward and then retracted to the original position without any forward locomotion;
- self-grooming, rubbing the body with paws or mouth and rubbing the head with paws.

### Beam Walking Test

Motor coordination and balance were evaluated by the ability of mice to traverse two narrow beams of different diameter to reach an enclosed safety platform [47]. Beams were horizontal and 50 cm above the table. Mice were trained to traverse a beam of 2 cm in diameter and then of 1 cm in diameter. Latency to traverse each beam and latency to fall were recorded.

### Hind-paw Footprint Test

This test was used to compare the gait of mutant mice with that of wild type control mice [48, 49]. To obtain footprints, the hind- and forefeet of the mice were coated with black and green nontoxic paints, respectively. The animals were then allowed to walk along a 50-cm-long, 10-cm-wide runway (with 10-cm-high walls) into an enclosed box and a sheet of white paper was placed on the floor of the runway. To characterize the walking pattern of each mouse we measured the average distance between each stride (stride length, SL) and the distance between left and right hind footprints (hind-base width, HB) and left and right front footprints (fore-base width, FB), respectively (all measured in millimeters).

### Statistical Analysis

Data were analyzed using parametric analysis of variance (ANOVA) with “genotype” (wild type and Tg) and “gender” (male and female) as between-subjects factors and “trial” and “time” as within-subject repeated measures, when appropriate (Beam Walking, Rotarod, Open Field). Post hoc comparisons were performed using the Tukey’s test. Statistical analysis was performed using Statview II (Abacus Concepts, CA, USA). Data are expressed as mean + SEM. A significance level of 0.05 was chosen.

## Results

### Immunoistochemical and biochemical characterization of the Tg-mouse

**Fig 1:**
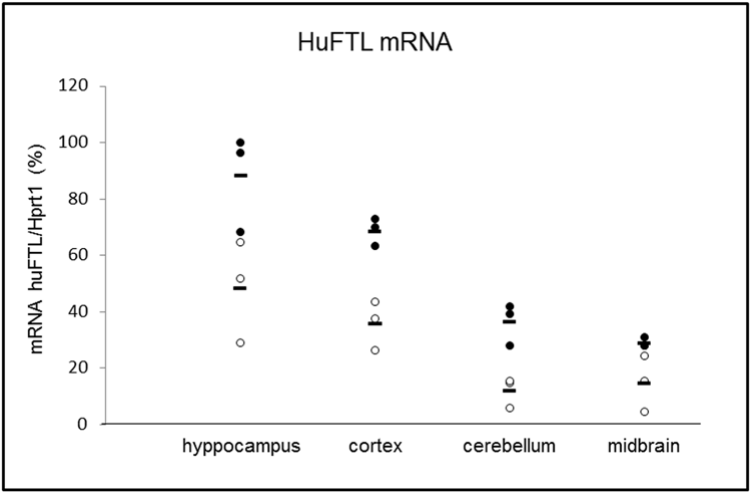
Evaluation of exogenous human FTL mRNA in the indicated areas of the brain of 8 month-old Tg-mice. Solid circles for males and the empty ones for females. The horizontal lines are the means. Data obtained by quantitative reverse-transcription polymerase chain reaction (qRT-PCR). The transcript of the transgene was higher in males than females, and higher in the hippocampus > cortex > cerebellum > midbrain.

The initial analyses performed on the different areas of the brain of Tg-mice showed that the expression of the transgene transcript was higher in the hippocampus compared with cortex, cerebellum and midbrain (Fig. 1).

**Fig 2:**
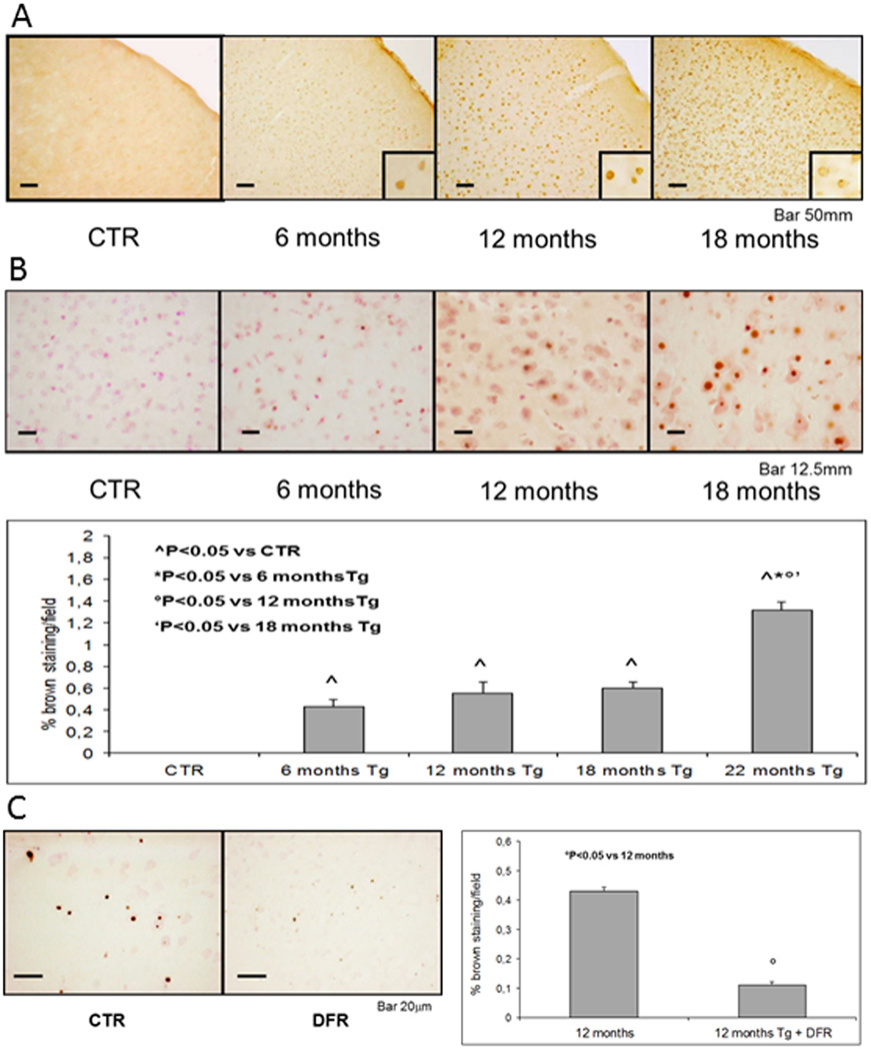
Ferritin and iron accumulation in the brain of Tg-mice. (A) Paraffin-embedded brain sections (40 *μ*m thick) of Tg and control mice were stained with a specific antibody for human L-ferritin chain. (B) Slices stained with DAB-enhanced Perl’s reaction showed a strong accumulation of ferritin/iron bodies in Tg-mouse brains. The quantitative evaluation of the percentage of brown staining in various fields showed the increase of the number and size of the granules with aging. At the optical microscope these granules seemed to be present mostly in neurons, and in the center of the cells in a nuclear/paranuclear position. (C) Mice were treated for 3 weeks with oral iron chelator Deferiprone (DFR) 1 mg/ml in drinking water ad libitum to reduce iron burden. The treatment reduced inclusion bodies in the brain both in number and in size.

Immunohistochemical analysis confirmed the high expression of the transgene and showed that its concentration increased with aging (Fig. 2A), in parallel with the endogenous ferritins (data not shown). The new transgenic mouse model confirmed the presence of ferritin/iron bodies in most tissues, principally in the brain: the main hallmark of the ferritinopathy patients and of the reported ferritinopathy mouse model [38, 40]. The inclusions became detectable with DAB-enhanced Perl’s reaction in 6-month-old mice, then the number and size of the granules increased with age. To have a quantitative evaluation of the iron bodies, we measured the percentage of brown staining area in various fields (Fig. 2B).

Because the deposits in the brain are Perl’s positive and are similar to hemosiderin, and hemosiderin can be solubilized in conditions of systemic iron deficiency, the treatment with Deferiprone, an oral iron-chelator, could reduce iron load in transgenic mice. Three weeks-Deferiprone-treatment reduced not only serum iron but also the number and sizes of the iron positive granules (Fig. 2C).

### Effect of neurotoxicant drugs on the Tg-mouse in FVB background

**Fig 3:**
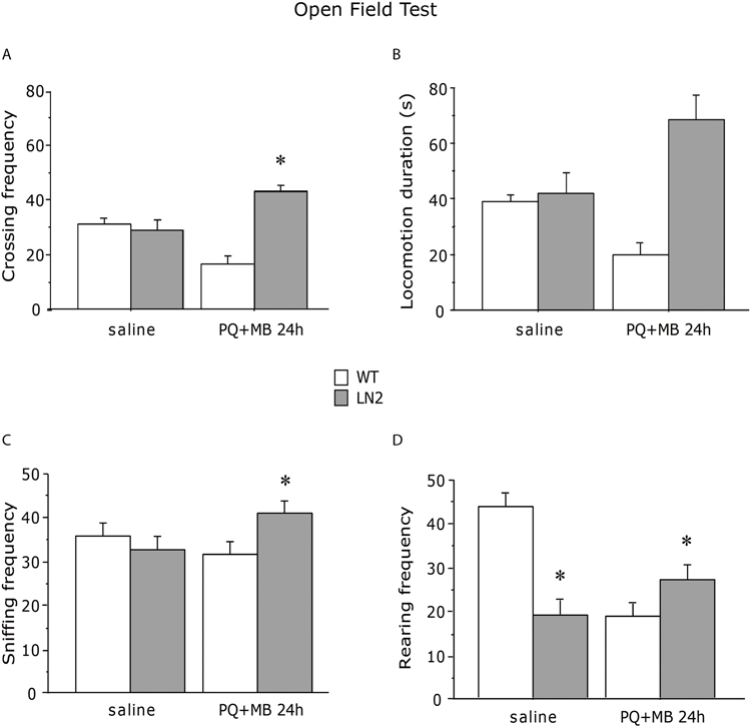
Assessment of spontaneous locomotor activity 24 h following administration of PQ+MB in old mice (21 month-old). After treatment Tg-mice showed increased locomotor (A, B) and exploratory activities (C, D), compared to wild type subjects of the same age. Results are presented as mean + S.E.M. * *P* < 0.05.

The Tg-mice in the FVB background did not show remarkable phenotypes and behaved similarly to the wt mice in behavioral tests. Thus, they were treated with herbicides, Paraquat and Maneb to induce neurodegenaration. The treatment resulted in an unexpected increase in locomotor and exploratory activities in the Tg-mice compared to wild-type littermates, as indicated by *crossing* (interaction between genotype and treatment F(1, 8) = 6.670; 3.998; P = 0.0325; 0.0806, respectively for frequency and duration) (Fig. 3A, 3B), *sniffing* (interaction between genotype and treatment F(1, 8) = 5.012, P = 0.05) (Fig. 3C) and *rearing* (interaction between genotype and treatment F(1, 8) = 11.310, P = 0.0099) (Fig. 3D).

### Motor coordination and balance of the Tg-mouse in C57BL/6 background

**Fig 4:**
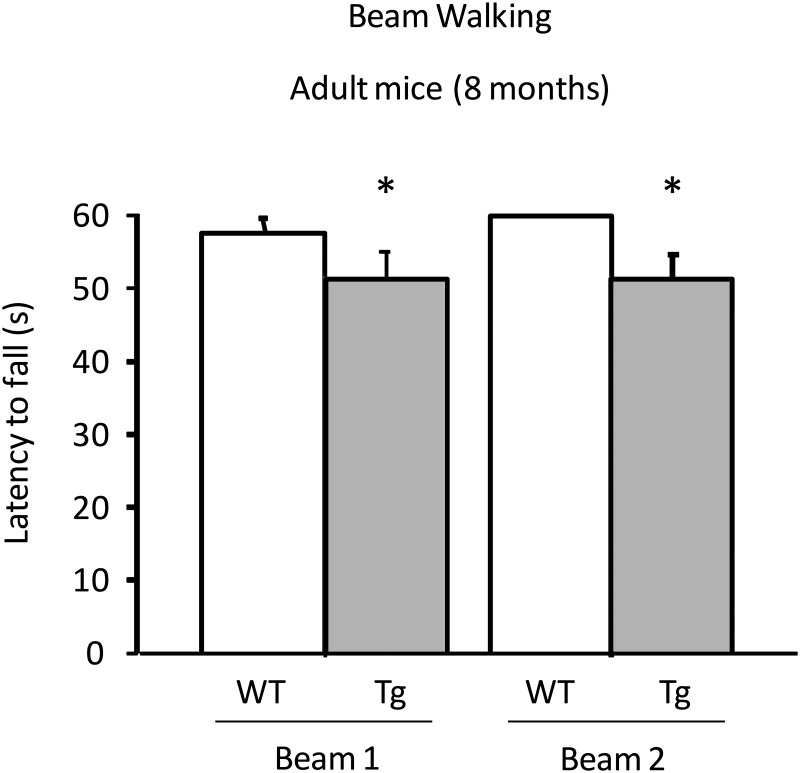
Assessment of motor function using the Beam Walking test. Tg adult mice (8 month-old) showed a lower latency to fall from the beams compared to wild-type. Results are presented as mean + S.E.M. * *P* < 0.05.

**Beam Walking Test**. Tg adult mice showed a lower latency to fall from the beams compared to wild-type (main effect of genotype F(1, 53) = 5.797, P = 0.0196) (Fig. 4), female mice showing an even shorter latency (interaction among trial, genotype and gender F(1, 53) = 5.701, P = 0.0206). No difference emerged in the performance between transgenic and wild-type mice at young (no main effect of genotype F(1, 26) = 0.401, P = 0.5319) and old (no main effect of genotype F(1, 17) = 2.305, P = 0.1473) age. At 8-month one wt male mouse found to be outlier and was therefore discarded from the analysis (Grubbs’ test performed by GraphPad Software). At 18 months, a main effect of gender also appeared, female mice were more able to stay on the beam without falling, compared to males, although this effect did not interact with genotype (main effect of gender F(1, 17) = 6.005, P = 0.0254).

**Fig 5:**
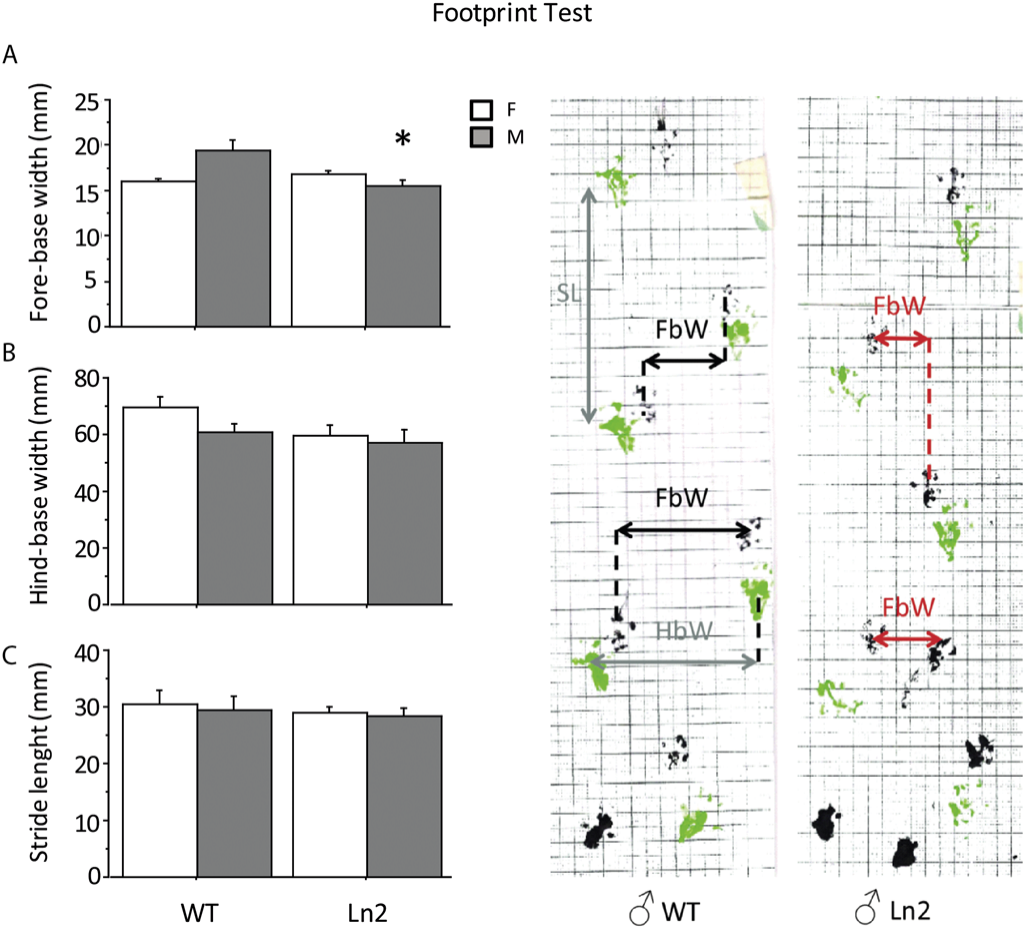
Assessment of gait using Footprint test. Tg old male mice (18 month-old) showed a significantly reduced fore-base width compared to controls **(A)**. Results are presented as mean + S.E.M. * *P* < 0.05.

**Hind-paw Footprint Test**. In order to confirm the motor phenotype of the mutants as a progressive movement disorder, adult and old mice were tested in the Footprint test to assess gait. In the 18-month-old group Tg male mice displayed an abnormal pattern in the fore-base width (interaction between genotype and gender F(1, 13) = 8.451, P = 0.0122) (Fig. 5A). No significant differences were found in this age group between wt and Tg-mice when measuring hind-base width HB (no main effect of genotype F(1, 13) = 0.379, P = 0.5490) (Fig. 5B) or stride length SL (no main effect of genotype F(1, 13) = 3.164, P = 0.0986) (Fig. 5C). No difference was found in the adult groups (no main effect of genotype F(1, 13) = 0.039; 3.039; 2.488; P = 0.8469; 0.1005; 0.1343 respectively for stride length, fore-base width and hind-base width measurements). Unfortunately, we could not use all the recorded footprints for the statistical analysis since, as it often happens, some mice retrace their own steps, making their paths unclear and hardly interpretable. Indeed, only 4–5 footprints in each group were sufficiently clear to be analyzed.

## Discussion

Neuroferritinopathy is caused by 9 types of mutation in the FTL gene, the most common is the 460InsA, with more than 40 patients described [25]. The other mutations were found in isolated cases and could not be followed in detail [14]. Animal models of the disorder are helpful to characterize the disease and to try therapeutic approaches. To this aim we studied an animal model expressing the pathogenic human FTL mutant 498InsTC under the phosphoglycerate kinase (PGK) promoter [40]. Notably, this mutant expresses a transgene similar to the one described by Vidal’s laboratory [38]. Both animal models were characterized by the formation of iron bodies in the brain and other tissues, which increased in number and size with age. These bodies, and their time dependent accumulation in the brain, represent the hallmark of the disorder, a finding that confirms that these are proper models of neuroferritinopathy.

Initial studies were performed in FVB background, the mouse strain normally used for transgene production. Even after ageing up to 2 years, no locomotor differences could be detected from the comparison with control littermates, possibly because the FVB background is known not to be adequate for behavioral studies. Several risk factors, such as environmental neurotoxicant exposures, have been hypothesized to increase the risk of developing neurodegenerative disorders, possibly including neuroferritinopathy. To test this hypothesis, Tg and control aged mice were treated with the herbicide Paraquat and the fungicide Maneb. These compounds have been previously shown to cause damage of the nigrostriatal system and to lead to neuronal death [50–52]. Although in most studies these herbicides are administered for a long period of time, because of their toxicity, we administered them only via an acute administration. Interestingly, while no major motor deficits emerged, 24 hours following the first treatment, the Tg-mice showed increased locomotor and exploratory activities compared to control subjects. These initial data suggest that adding up the brain iron accumulation with additional oxidative stress caused by PQ+MB treatment, in aged mice might result in a paradoxical behavioral activation. Prolonged elevation of ferritin levels within dopaminergic midbrain neurons results in their progressive age-related neurodegeneration [53]. This is initially manifested as a striatal axonopathy accompanied by loss in locomotor function and is followed by a significant loss in striatal dopamine content and subsequent dopaminergic cell loss in the substantia nigra [53]. Oxidative stress, such as that caused by exposure to herbicides, might interact with the pathological consequences of iron accumulation leading to a peculiar motor phenotype such as that previously described. Indeed, oxidative stress has been shown to alter normal dopaminergic neurotransmission [54] and to increase dopaminergic activity [55] inducing relevant adaptive responses of dopaminergic receptors in specific brain regions [56]. It is also demonstrated that stress manipulations induced the alteration in motor activity of experimental animals, and dopaminergic pathways are crucial to neural substrates for the control of spontaneous locomotor activity [57]. It is thus possible to hypothesize that accumulation of ferritin/iron might lead to a paradoxical increase in dopaminergic activity, suggesting a “positive” feedback on the nigrostriatal system. It would be important to assess, in future studies the long-term effects of exposure to toxicants and their interaction with the pathological phenotype characterizing NBIA.

The C57BL/6 strain is better suited for behavioral studies, thus we backcrossed the original FVB mouse strain for 7 generations in C57BL/6J. We thought important to assess behavioral abnormalities well before the occurrence of neurodegeneration since they might represent precocious hallmarks of the pathology, before the clinical phenotype is observed. Thus, wild-type and transgenic animals were studied at different ages to assess motor function in a battery of tests commonly used to this purpose, such as Beam Walking and Footprint tests. Different subjects were used at different ages to avoid carry−over effects of previous testing. Results for motor coordination using the Beam Walking test indicate that an effect of the genotype emerges at adult age (8 months) with an inability of the Tg-mice to traverse a beam compared to wild-type, while no difference was found at younger or older age. This might be explained by the fact that, while in young subjects the phenotype is not yet evident, this might be masked in the old group by their tendency to be immobile, thus not traversing the pole at all.

In order to characterize more quantitatively differences in gait, the Footprint test was used and an abnormal gait pattern was found in the old male Tg-mice. The impairment in this specific measure suggests a profound motor deficit caused by an inability of the animal to successfully extend forepaws. Abnormal gait has been referred to impaired cerebellar motor or peripheral neurological function. Although we did not find significant differences in the stride length, it is important to consider that the number of subjects was necessarily low as in this test some subjects had to be discarded because they often walk backward on their own footsteps thus making it very difficult to assess the walking path.

Overall, data confirm that transgenic mice display a number of motor abnormalities reminiscent of those occurring in neuroferritinopathy, including locomotor abnormalities [14]. However the ones we observed in our Tg-mice were less severe than those described by the group of Vidal [38]. We were able to identify the time windows associated to disease phenotype manifestation. In particular, we found a significant decrease in motor performance at adult age (8 months). Motor deficits progressively worsened with age, 18 months-old Tg-mice showing gait abnormalities. As for gender differences, interestingly we found that at adulthood (8 months) female Tg-mice revealed a poorer motor performance than males, while in the old age this was totally reverted. These data are in line with a large body of evidence indicating gender differences in the rate of aging, with females overall showing healthier phenotypes as they become older [58]. This effect might have partially masked the pathological phenotype in the aged subjects and indicates that attention must be paid to gender x genotype effects in these pathologies. Numerous biological mechanisms could underlay these effects, as an example, sex hormones such as estrogen might influence brain aging attenuating the age-related decline in female subjects [59].

Neuroferritinopathies are key examples of pathologies in which brain iron dysregulation is tightly associated with neurodegeneration. Since iron accumulation is also observed in Parkinson and Alzheimer’s disease, and in other less common degenerative disorders, the clarification of the connection between iron dysregulation and neurodegeneration may have an important impact in understanding the basic mechanisms underlying neurodegenerative processes.

Overall, data presented in this paper suggest that this transgenic mouse model, expressing the pathogenic mutant human ferritin light chain FTL498–499InsTC, can be used as a powerful tool to investigate the pathogenic mechanisms underlying neuroferritinopathy. Although this is a first behavioral characterization, further studies on this mouse model might help elucidating the mechanisms underlying the pathological phenotype and guide the development of pharmacological tools to counteract it.
